# A novel surgical strategy of three column osteotomy at non-lesioned area for correcting severe angular kyphosis due to Pott’s disease: a retrospectively study

**DOI:** 10.1038/s41598-023-48891-y

**Published:** 2023-12-04

**Authors:** Deng Zhao, Fei Wang, Zhengjun Hu, Rui Zhong, Huaqiang Huang, Zhong Zhang, Dengxu Jiang, Yan Liang, Yijian Liang

**Affiliations:** 1 Department of Orthopaedics, The Third People’s Hospital of Chengdu/The Affiliated Hospital of Southwest Jiaotong University, Chengdu, China; 2 Department of Spinal Surgery, Peking University People’s Hospital, Beijing, China

**Keywords:** Neuroscience, Neurology

## Abstract

Severe sharp angular kyphosis resulting from Pott’s disease typically necessitates surgical intervention. The deployment of three-column osteotomy within the lesion and apical regions has been validated as an effective modality for the amelioration of angular kyphosis. Nonetheless, a propensity for residual kyphosis persists, accompanied by a significant perioperative risk profile. In pursuit of optimizing correctional outcomes and diminishing complication rates, we proposed an innovative surgical approach, utilizing osteotomy in the non-lesioned zones for the rectification of severe angular kyphosis associated with Pott’s disease. This retrospective investigation encompasses 16 subjects who underwent this novel surgical tactic, involving osteotomies in non-lesioned vertebral segments, at our institution from 2016 to 2018. Radiographic measures, encompassing kyphotic angle and sagittal vertical axis (SVA), were documented at baseline and during terminal follow-up. Neurological status was evaluated via the American Spinal Injury Association (ASIA) grading system. Operative duration, volume of hemorrhage, and perioperative complications were systematically recorded. The cohort included 6 males and 10 females with an average age of 30.7 ± 11.41 years. Follow-up intervals spanned 24 to 42 months. Mean operative time and blood loss were 492 ± 127.3 min and 1791 ± 788.8 ml, respectively. The kyphotic angle improved from 97.6 ± 14.6° to 28.8 ± 18.70°. In cases with lumbar afflictions, vertebral restoration was achieved (L1–L5 and L2–S1). Initial mean SVA of 6.7 ± 3.58 cm was reduced to 3.3 ± 1.57 cm at follow-up. Neurological function enhancement was observed in six patients, while ten maintained baseline status. Complication rates, including wound infection and rod fracture at 12 months, were observed in approximately 11.8% of cases. Our findings suggest that the surgical strategy is both effective and safe for addressing severe angular kyphosis due to Pott’s disease, contingent upon the expertise of the surgical unit.

## Introduction

Spinal tuberculosis, commonly referred to as Pott’s disease, was first delineated by Percival Pott in 1779^1^. While it afflicts a minority—less than 2%—of individuals with tuberculosis, its incidence is escalating^2^. The disease predominantly targets the anterior column of the vertebral body, often resulting in its collapse and subsequent kyphosis. Research demonstrates that nonsurgical management frequently culminates in kyphosis, with an average deviation of 15°, and in 3–5% of patients, this curvature may exceed 60°^3^. The risk and magnitude of kyphosis are notably more severe in cases contracted during childhood^4^. Complications of advanced kyphosis include low back pain, compromised pulmonary function, and risk of spinal cord injury. Existing literature indicates a correlation between severe kyphosis and spinal cord injury, with incidence rates between 10 and 43%^5,6^.

**Figure 1:**
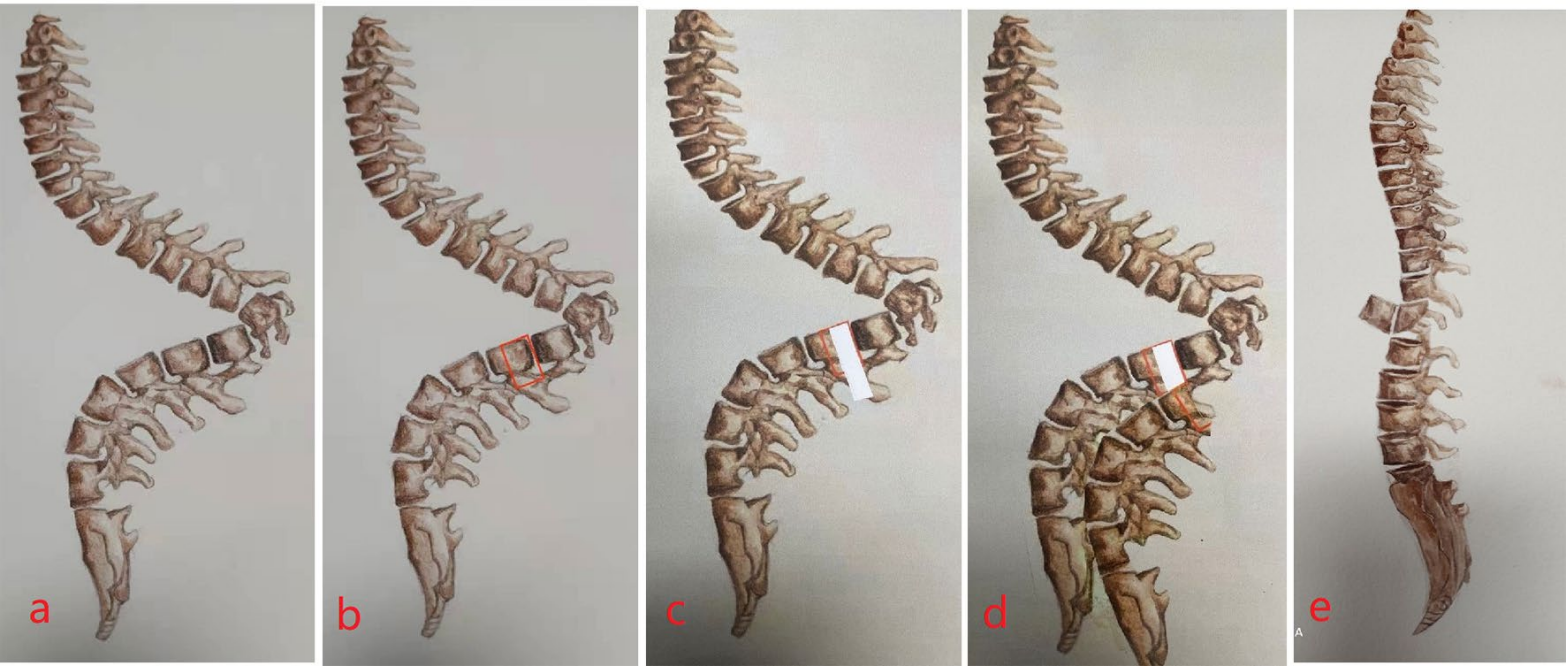
Schematic of the procedure. Panel (**a**) depicts the presence of severe sharp angular kyphosis. In panel (**b**), the vertebral segment immediately subapical to the distal apex is identified for intervention. Panel (**c**) illustrates the execution of vertebral column resection (VCR) on the chosen vertebra. Subsequently, panel (**d**) demonstrates the incremental retraction and elevation of the osteotomized vertebral ends. Finally, panel (**e**) presents the resultant correction of the overarching compensatory curve and the resolution of kyphosis. (Adapted from Liang Y, et al. Vertebral column resection (VCR) at the subapical vertebra for correction of angular kyphosis associated with neurofibromatosis type 1 (NF1): a case report. Eur Spine J. 2022).

Surgical intervention for Pott’s disease-related kyphosis is aimed at mitigating spinal cord compression and alleviating low back pain, thereby striving to improve respiratory and neurospinal functionality, and ultimately patient quality of life^7^. The prevailing surgical options, posterior 3-column osteotomies such as posterior vertebral column resection (PVCR) and pedicle subtraction osteotomy (PSO), are usually performed at the apex of the deformity^7^. However, dense adhesions around the spinal cord at the tuberculous lesion's apex may jeopardize the cord's vascular supply and compress adjacent nerve roots, presenting substantial risks, including excessive hemorrhage, neural trauma, and potential for both anterior column fusion failure and long-term internal fixation failure^8^. To circumvent these risks, our team has innovated an osteotomy technique that strategically avoids the apical region^9^ (Fig. 1), a method previously utilized in addressing severe scoliosis secondary to neurofibromatosis.

In the current investigation, we employed this novel technique in patients with spinal tuberculosis-induced angular kyphosis. Osteotomies were executed in healthy vertebral units adjacent to the lesions. The disease process had precipitated the collapse of multiple vertebrae, engendering severe angular kyphosis and the development of compensatory curvatures, thereby disrupting the spine's physiological alignment. The selected osteotomy site was the unaffected vertebra immediately adjacent to the lesioned segments. Herein, we delineate the therapeutic outcomes and collate clinical data from patients subjected to this osteotomy regimen for the management of kyphosis stemming from spinal tuberculosis, with the objective of elucidating a superior treatment strategy.

## Materials and methods

### General patient information

Prior to study commencement, approval was secured from the Institutional Review Board (IRB approval number: 2022-S-23). Written informed consent was obtained from all participants for the inclusion in the study and any associated investigative procedures. The cohort comprised patients diagnosed with angular kyphosis secondary to healed spinal tuberculosis, who received treatment via osteotomy in non-lesioned segments, augmented by bone graft fusion and pedicle-screw fixation, between 2016 and 2018 at our institution.

Eligibility for inclusion required fulfillment of several criteria: (1) confirmation of healed spinal tuberculosis with concurrent angular kyphosis; (2) cases demonstrating neurological deficits or severe femoroacetabular impingement syndrome; (3) presence of five or more compromised vertebral bodies; and (4) a minimum postoperative follow-up duration of 24 months. Conversely, exclusion criteria precluded: (1) patients manifesting active tuberculosis within extraspinal sites; (2) those with comorbid pulmonary pathology; and (3) individuals who had not been subjected to posterior 3-column osteotomy procedures.

### Surgical methods

Following the induction of general anesthesia and endotracheal intubation, patients were positioned prone. A posterior midline incision was made, extending across the lesions and planned osteotomy region.

Instrumentation levels were determined by the pathological anatomy and the overall sagittal balance; the lower instrumented vertebra (LIV) was set at a minimum of three segments below the osteotomy, while the upper instrumented vertebra (UIV) was typically T1, influenced by the patient’s sagittal profile and vertebral involvement. Owing to vertebral collapse, the standard configuration involved an angular kyphosis with two secondary lordotic curves.

The operative team exposed the laminae bilaterally to the facet joints' lateral edges. Pedicle screws were then inserted above and below the affected and osteotomy regions according to the preoperative design. Correct placement was ascertained using fluoroscopy before positioning a precontoured titanium rod across the screws on the side contralateral to the surgeon, establishing temporary stabilization.

Laminectomy was performed on the designated vertebra and adjacent segments using an ultrasonic osteotome. In lumbar segments, bilateral transverse processes were resected at their bases. For thoracic segments, the transverse process, medial rib, and rib head were also removed to fully detach the osseous structures from surrounding soft tissues. Hemostasis was achieved using gauze packing post-resection.

On the contralateral side of the initial temporary fixation, a V-shaped osteotomy was created using an ultrasonic osteotome, extending from the pedicle to the anterior vertebral wall. The resected vertebral segment was then progressively enlarged to remove the superior discs. After securing a temporary rod, osteotomy was completed by removing the remaining bone on the opposing side.

The spinal deformity correction commenced with the insertion of a titanium rod, pre-bent to a lesser degree of curvature than the kyphotic angle, onto the left pedicle screws. Subsequent relaxation of the right-side temporary rod permitted the use of the pre-bent rod's tension for initial correction. Following the placement of the contralateral rod, the procedure involved alternating adjustments of the rods until satisfactory correction of the kyphosis was attained.

**Figure 2:**
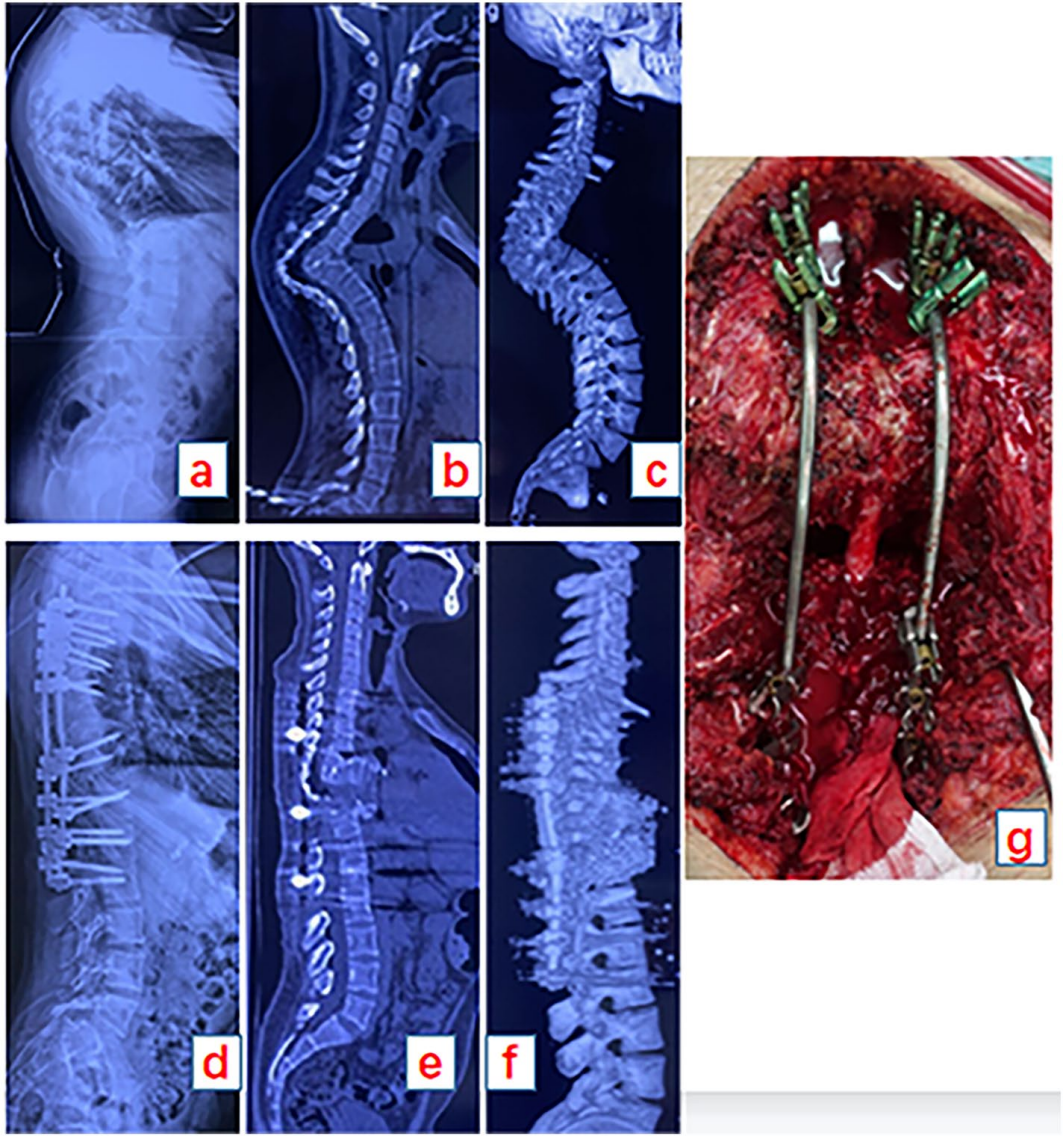
21-year-old male patient presented with severe sharp angular kyphosis caused by Pott’s diseaset with the tuberculosis lesion extending from T4 to T9. Panel (**a**) illustrates a lateral radiograph of the entire spine, while panel (**b**) reveals the computed tomography (CT) image, indicating partial correction of the compensatory curve following six months of halo-pelvic traction, yet the pronounced angular kyphosis persists. Panel (**c**) provides a three-dimensional (3D) CT reconstruction, highlighting the severity of the angular kyphosis. Panels (**d**, **e**, and **f**) display postoperative lateral radiographs and CT scans demonstrating that targeted osteotomy at T10 yielded satisfactory realignment, with the acute angular deformity addressed. Finally, panel (**g**) presents an intraoperative photograph showcasing the spinal cord at T10 subsequent to a 3-column osteotomy.

Subsequent to the initial correction, the process entailed alternating the application of force via the titanium rods on the left and right, thereby incrementally rectifying the kyphotic curvature. This stepwise correction was conducted with diligence to ensure the structural integrity of the spine. Following each incremental adjustment, the dural sac's tension was examined, with decompression being augmented as indicated. Additionally, to facilitate sagittal realignment, an assistant manipulated the patient's lower extremities and pelvis, when deemed necessary, to induce a distal posterior rotational effect, as depicted in Fig. 2. Throughout the surgical intervention, continuous neuromonitoring via somatosensory evoked potentials (SEP) and motor-evoked potentials (MEP) was employed to ensure the preservation of neurological function.

### Postoperative follow up and data collection

Radiographic assessment of the entire spine was performed prior to patient discharge and during subsequent visits at 3, 6, and 12 months, as well as at 2 years and the ultimate evaluation point. Collected data encompassed the degree of lesion degradation, kyphotic angles prior to and subsequent to surgery (ascertained with consistent reference to the upper and lower vertebrae, excluding the apex region), as well as the sagittal vertical axis (SVA) values before and after the intervention, in addition to the assessment of postoperative internal fixation integrity.

### Statistical analysis

Data analysis was executed utilizing the SPSS software, version 20.0 (IBM Corp., Armonk, NY). Imaging measurements obtained preoperatively and postoperatively are delineated as the mean ± standard deviation. Statistical significance was determined through the paired t-test, with a p-value of less than 0.05 indicating significance. The STROBE guidelines informed the design and execution of this investigation.

## Results

The study included 16 patients, with a composition of six males and ten females, meeting the inclusion criteria. The mean age was calculated to be 30.7 ± 11.41 years, with a range of 21 to 61 years. All participants had a history of spinal tuberculosis contracted in childhood. In 14 instances, the tubercular lesions involved both thoracic and lumbar vertebrae, whereas in two instances, only lumbar vertebrae were implicated. The affected vertebrae averaged 7.3 ± 2.11, with a range of 5 to 10 vertebrae. Halo-pelvic traction was applied in 11 patients to mitigate kyphosis; nonetheless, the intervention yielded limited improvement in angular kyphosis over an average duration of six months. The mean preoperative kyphotic angle was 97.6 ± 14.6°, with a span from 76° to 121°. The mean preoperative SVA was recorded at 6.7 ± 3.58 cm, ranging from 2.7 to 11.6 cm (refer to Table 1).

**Table 1:** Pre- and Post-operative radiological parameters.

Lesion area	cases	Number of vertebrae involved	SVA		Angle of kyphosis		
			Pre-op	Post-op			
Thoracic and lumbar spine	14	7.8 ± 1.8	6.7 ± 3.58 cm	3.3 ± 1.57 cm	Pre-op	Post-op	P
					101 ± 13.4	28.5 ± 9.1	< 0.01
Lumbar spine	2	5			Pre-op	Post-op	P
					80.5 ± 4.5	− 11.5 ± 1.5	< 0.01

*SVA* sagittal vertical axis; P < 0.05 was considered statistically significant.

Neurological status was assessed employing the ASIA Impairment Scale. The distribution included nine ASIA D cases and seven ASIA E cases, as indicated in Table 2.

**Table 2:** Pre- and post-operative ASIA Grade.

ASIA Grade	Pre-op	Post-op
D	9	3
E	7	13

*ASIA*, American Spinal Injury Association.

**Figure 3:**
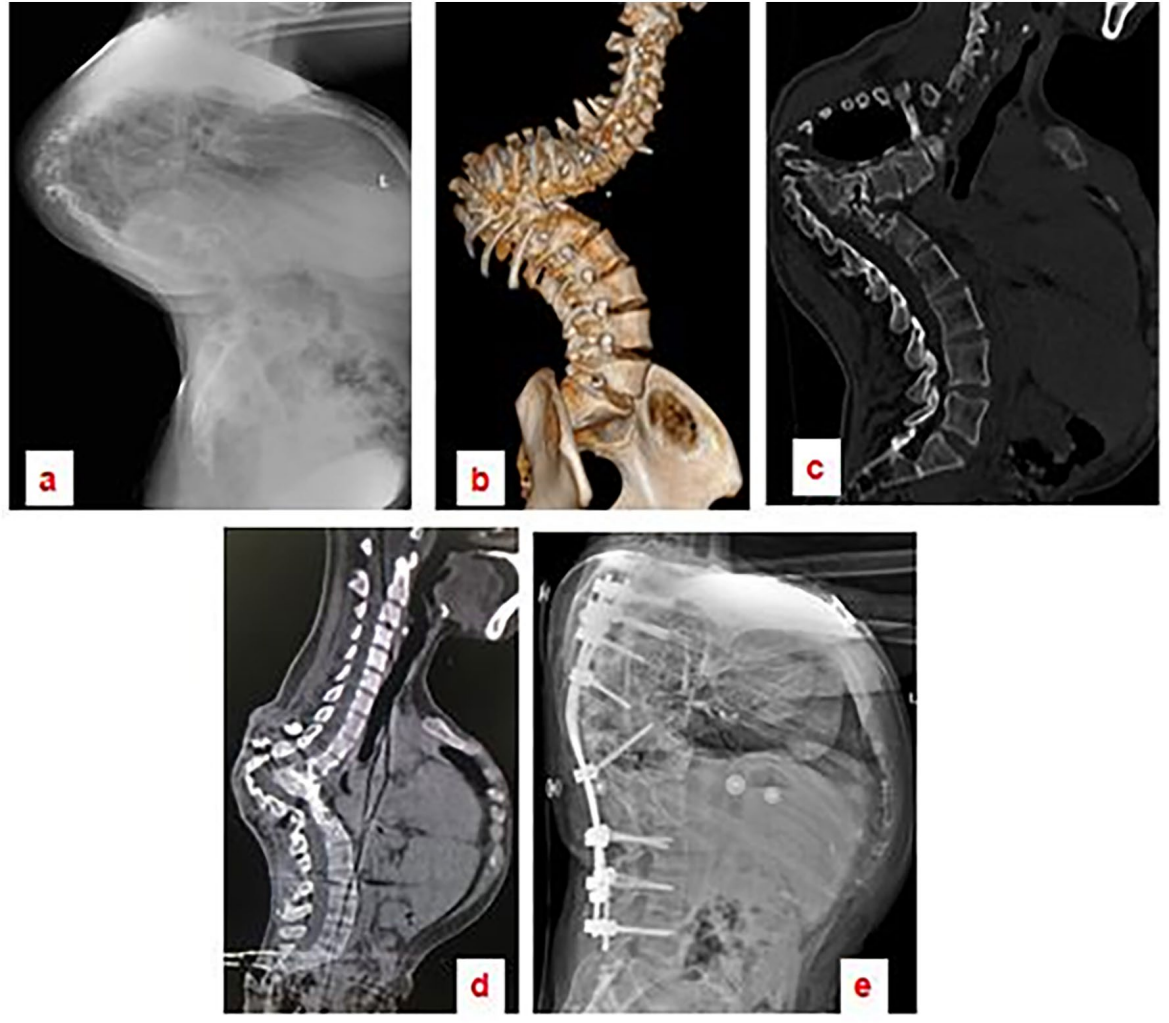
29-year-old female patient with severe sharp angular kyphosis due to Pott’s disease. The tuberculosis lesion extended from T4 to T12. Image (**a**) is a lateral radiograph of the entire spine. Images (**b** and **c**) are 3D computed tomography (CT) scans illustrating the pronounced sharp angular kyphosis. Image (**d**) demonstrates that, following six months of halo-pelvic traction, there was a notable correction of the compensatory curve; however, the sharp angular kyphosis persisted. Image (**e**) is a postoperative lateral radiograph showing that a satisfactory correction was realized after performing an osteotomy at the L1 level.

**Figure 4:**
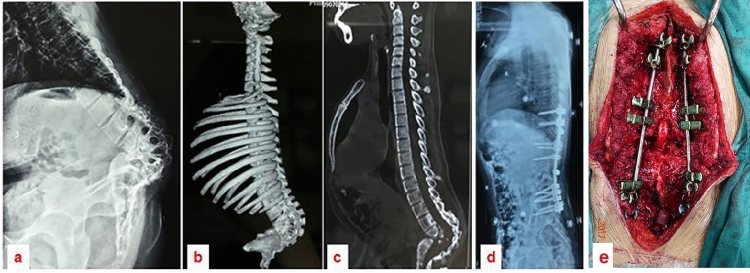
27-year-old female patient with severe sharp angular kyphosis due to Pott’s disease. Image (**a**) shows a lateral radiograph of the entire spine. Images (**b** and **c**) display 3D CT scans of the spine, highlighting the sharp angular kyphosis affecting the lumbosacral region. Image (**d**) is a postoperative lateral radiograph in which satisfactory correction is evident subsequent to an osteotomy at T12. Image (**e**) exhibits an intraoperative view of the spinal cord at T12 following a three-column osteotomy.

The surgical procedure averaged 492 ± 127.3 min in duration, with blood loss averaging 1791 ± 788.8 ml (range: 800–3000 ml). The follow-up period lasted 31.2 ± 5.49 months (range 24–42 months). The mean kyphotic angle experienced a substantial reduction postoperatively, descending from 97.6 ± 14.6° to 28.8 ± 18.70°, which constitutes a 65% correction rate. The lesions were localized to the lumbar spine in two patients, with a span from L1 to L5 in one case and from L2 to S1 in another. For the remaining patients, thoracic spinal lesions were identified. A three-column osteotomy was performed on the vertebra located below the apical region in 14 cases (Fig. 3), and above the apical region in two cases (Fig. 4). The respective preoperative kyphotic angles of these two patients were 85° and 61°, with postoperative lordosis angles recorded at 13° and 10°, respectively (Time scale analysis: − 13 and − 10). Post-surgical SVA readings indicated an improvement from 6.7 ± 3.58 to 3.3 ± 1.57 cm, effectively restoring the overall sagittal alignment of the spine, as detailed in Table 1. Neurological function was enhanced in six patients, whereas no substantial change was noted in the remaining ten at the final evaluation (Table 2).

During intraoperative neuromonitoring, one patient exhibited a marked decrease in somatosensory evoked potentials (SEP) and manifested temporary neurological deficits postoperatively. The patient’s condition improved to baseline following a regimen of exercise-based rehabilitation by the final follow-up. Additionally, one individual developed an infection at the surgical site, which was successfully managed with incision debridement. There was also a case of rod fracture within the internal fixation apparatus at the 12-month mark post-surgery, ascribed to insufficient anterior column fusion. Consequently, revision surgery was necessitated.

## Discussion

This study yielded a kyphosis correction rate of 65% among the cohort. Prior to surgical intervention, nine patients exhibited various levels of spinal cord injury. Postoperatively, a 66.7% improvement in neurological function was observed, affirming the efficacy of the novel osteotomy technique in non-lesioned areas for achieving substantial kyphosis correction and significant spinal decompression. Only a single patient experienced transient neurological decline post-surgery, which was subsequently ameliorated through active rehabilitation and exercise. This suggests that, while realignment of the spinal and spinal canal may exert tensile stress on spinal nerve roots of both anterior and posterior columns, leading to potential strain on the spinal cord, gradual exercises can facilitate the recovery of spinal cord function. No further exacerbation of neurological deficits was noted in the patient group.

The administration of anti-tuberculosis drugs has enhanced the management of spinal tuberculosis in regions with robust medical infrastructure. However, the prevalence of the condition remains elevated in areas with suboptimal medical services^10^. Untreated spinal tuberculosis carries significant risks due to its predilection for the spine, potentially leading to deformities, respiratory compromise, and neurological sequelae^5,6,11^. The progression of kyphosis in spinal tuberculosis is delineated into two distinct phases: the active and the healed stages of the infection. In adults, kyphotic deformity typically develops during the active phase and ceases once the infection resolves. In contrast, pediatric patients may experience progression during both stages, resulting in more pronounced deformities^4,12^. Evidence suggests that early-onset disease, particularly before the age of seven, coupled with infection spanning more than three thoracic or thoracolumbar vertebrae, markedly escalates the risk of progressive kyphosis. Thus, early surgical management is advocated in such scenarios^13^. Compensatory changes occur above and below the lesioned vertebrae, creating long-arc lordotic curves to maintain spinal stability. At the kyphosis apex, the spinal cord may be significantly elongated, heightening the risk of neurological compromise^14^. Neurological complications, including during the quiescent phase of tuberculosis, can manifest due to compression from kyphotic deformity, osseous bridges, and fibrosis, potentially culminating in paraplegia^15,16^. The current investigation concentrated on individuals with a history of pediatric spinal tuberculosis that resulted in extensive vertebral body destruction and fusion, leading to severe kyphotic deformities. Consequently, the surgical approach entailed elevated risks, presenting a formidable challenge to the treating physicians. Notably, 56.2% of the patients had discernible neurological impairments preoperatively.

The most efficacious surgical intervention for severe kyphosis secondary to spinal tuberculosis is identified as the three-column osteotomy. Currently, surgeons have three procedural options: the anterior approach, a combined anterior and posterior approach, and exclusively the posterior approach^7,17^. The anterior approach encounters increased complexity and a heightened risk of complication when managing kyphotic angles surpassing 60°, attributable to fibrotic tissue and adhesions within the affected zone. Resection via the anterior route may induce temporary excessive tension on the spinal cord, elevating the risk of consequential neurological impairments. Additionally, the amalgamation of anterior and posterior surgical techniques is frequently necessitated to realize optimal postoperative outcomes, particularly in cases involving bony fusion within the posterior column. Hence, the posterior approach is predominantly utilized for the rectification of severe kyphotic deformities caused by spinal tuberculosis^7,18^.

Prior research has documented that posterior 3-column osteotomies are capable of correcting deformities between 39.2° and 88.4°, reducing spinal cord compression, and improving neurological function by 33–100%, with associated surgical complication rates of 8–20%^19^. The prevalent forms of posterior 3-column osteotomies currently include pedicle subtraction osteotomy (PSO), vertebral column resection (VCR), and vertebral column decancellation (VCD). The application of PSO in a single-segment capacity is efficacious in rectifying kyphotic deformities of 30–50° in non-tuberculosis-related cases^20^. It has been posited that in spinal tuberculosis, the region affected by angular kyphosis can be treated as a single vertebral entity, thereby allowing for decompression and corrective interventions akin to PSO, which have been shown to produce favorable outcomes^21,22^. PSO entails the removal of bone from the middle and posterior columns, with the anterior vertebral body wall acting as a fulcrum, analogous to the mechanism of closing wedge osteotomies. However, PSO may precipitate a significant decrease in spinal canal dimensions, increasing the potential for spinal cord damage in patients with tuberculous kyphotic afflictions^7^. VCR, while more demanding surgically, permits direct decompression of the spinal cord's anterior aspect, correction of the kyphotic curvature, and may mitigate the risk of spinal cord constriction associated with PSO. Thus, VCR is extensively employed in addressing kyphosis following the collapse of the anterior column^7,19^. Substantial evidence from multiple studies attests to the efficacy of VCR in the management of kyphotic distortion resulting from spinal tuberculosis^23–26^. VCD, on the other hand, has been recognized for its simpler execution relative to VCR, superior corrective capacity, and obviation of anterior column support materials, thereby diminishing the likelihood of surgical complications^27^.

This retrospective study evaluated the surgical rectification of severe angular kyphosis in 16 patients, a condition consequent to spinal tuberculosis. The literature advocates posterior 3-column osteotomies as the surgical intervention of choice for this type of kyphosis^7,14,19,26^. The cohort under investigation presented with angular kyphosis stemming from the destruction or collapse of multiple vertebral bodies (ranging from 5 to a maximum of 10). These cases were characterized by pronounced deformity, a spectrum of lesion extent, and significant local scar tissue with adhesions, alongside marked spinal cord compression. The presence of multiple spinal nerve roots in the posterior column posed additional constraints to the already limited surgical field. Direct osteotomy within the lesioned region significantly escalates the risk of surgical complications. Notably, spinal cord injury and extensive hemorrhage are recognized as the most severe complications associated with 3-column osteotomies^28,29^. To mitigate the increased surgical risks attributable to the pathological features of the lesions in patients with spinal tuberculosis, osteotomies were strategically performed in non-lesioned areas within this study's cohort. The approach involved executing an osteotomy on an intact vertebral body adjacent to the lesion, either superiorly or inferiorly, with the objective of realigning the spine and reconfiguring the spinal canal alignment, thereby facilitating spinal decompression.

In summary, osteotomy conducted in non-lesioned areas for angular kyphosis due to spinal tuberculosis effectively circumvents the intricate anatomical structures and reduces further aggravation of the already compressed spinal cord. This technique has demonstrated favorable orthopedic results and an increased margin of surgical safety.

## References

[1] Dobson J (1972). Percivall Pott. Ann. R. Coll. Surg. Engl..

[2] Iademarco MF, Castro KG (2003). Epidemiology of tuberculosis. Semin. Respir. Infect..

[3] Rajasekaran S, Shanmugasundaram TK (1987). Prediction of the angle of gibbus deformity in tuberculosis of the spine. J. Bone Joint Surg. Am..

[4] Rajasekaran S (2007). Buckling collapse of the spine in childhood spinal tuberculosis. Clin. Orthop. Relat. Res..

[5] Sai KNA, Vaishya S, Kale SS, Sharma BS, Mahapatra AK (2007). Surgical results in patients with tuberculosis of the spine and severe lower-extremity motor deficits: A retrospective study of 48 patients. J. Neurosurg. Spine..

[6] Tuli SM (1995). Severe kyphotic deformity in tuberculosis of the spine. Int. Orthop..

[7] Boachie-Adjei O, Papadopoulos EC, Pellisé F, Cunningham ME, Perez-Grueso FS, Gupta M (2013). Late treatment of tuberculosis-associated kyphosis: Literature review and experience from a SRS-GOP site. Eur. Spine J..

[8] Bridwell KH (2006). Decision making regarding Smith-Petersen vs pedicle subtraction osteotomy vs vertebral column resection for spinal deformity. Spine.

[9] Liang Y, Hu Z, Zhao D, Wang F, Zhong R (2022). Vertebral column resection (VCR) at the subapical vertebra for correction of angular kyphosis associated with neurofibromatosis type 1(NF1): A case report. Eur. Spine J..

[10] Global tuberculosis control (2010). key findings from the December 2009 WHO report. Wkly. Epidemiol. Rec..

[11] Rajasekaran S, Dilip S, Shetty AP, Kanna RM (2018). Spinal tuberculosis: Current concepts. Glob. Spine J..

[12] Rajasekaran S (2002). The problem of deformity in spinal tuberculosis. Clin. Orthop. Relat. Res..

[13] Rajasekaran S (2001). The natural history of post-tubercular kyphosis in children: Radiological signs which predict late increase in deformity. J. Bone Joint Surg. Br..

[14] Rajasekaran S (2012). Kyphotic deformity in spinal tuberculosis and its management. Int. Orthop..

[15] Jain AK, Kumar J (2013). Tuberculosis of spine: Neurological deficit. Eur. Spine J..

[16] Garg RK, Somvanshi DS (2011). Spinal tuberculosis: A review. J. Spinal Cord Med..

[17] Khanna K, Sabharwal S (2019). Spinal tuberculosis: A comprehensive review for the modern spine surgeon. Spine J..

[18] Jain AK, Dhammi IK, Jain S, Mishra P (2010). Kyphosis in spinal tuberculosis: Prevention and correction. Indian J. Orthop..

[19] Panchmatia JR, Lenke LG, Molloy S, Cheung KM, Kebaish KM (2015). Review article: Surgical approaches for correction of post-tubercular kyphosis. J. Orthop. Surg..

[20] Bridwell KH, Lewis SJ, Rinella A, Lenke LG, Baldus C, Blanke K (2004). Pedicle subtraction osteotomy for the treatment of fixed sagittal imbalance: Surgical technique. J. Bone Joint Surg. Am..

[21] Bezer M, Kucukdurmaz F, Guven O (2007). Transpedicular decancellation osteotomy in the treatment of posttuberculous kyphosis. J. Spinal Disord. Tech..

[22] Kalra KP, Dhar SB, Shetty G, Dhariwal Q (2006). Pedicle subtraction osteotomy for rigid post-tuberculous kyphosis. J. Bone Joint Surg. Br..

[23] Zhang HQ, Li JS, Liu SH, Guo CF, Tang MX, Gao QL (2013). The use of posterior vertebral column resection in the management of severe posttuberculous kyphosis: A retrospective study and literature review. Arch. Orthop. Trauma Surg..

[24] Hamzaoglu A, Alanay A, Ozturk C, Sarier M, Karadereler S, Ganiyusufoglu K (2011). Posterior vertebral column resection in severe spinal deformities: A total of 102 cases. Spine.

[25] Pappou IP, Papadopoulos EC, Swanson AN, Mermer MJ, Fantini GA, Urban MK (2006). Pott disease in the thoracolumbar spine with marked kyphosis and progressive paraplegia necessitating posterior vertebral column resection and anterior reconstruction with a cage. Spine.

[26] Sangondimath G, Mallepally AR, Chhabra HS (2019). Severe Pott's kyphosis in a 19-month-old child: Case report and review of literature. World Neurosurg..

[27] Wang Y, Lenke LG (2011). Vertebral column decancellation for the management of sharp angular spinal deformity. Eur. Spine J..

[28] Issack PS, Boachie-Adjei O (2012). Surgical correction of kyphotic deformity in spinal tuberculosis. Int. Orthop..

[29] Lenke LG, Sides BA, Koester LA, Hensley M, Blanke KM (2010). Vertebral column resection for the treatment of severe spinal deformity. Clin. Orthop. Relat. Res..

